# Effects of Pharmacologic Dose of Resveratrol Supplementation on Oxidative/Antioxidative Status Biomarkers in Nonalcoholic Fatty Liver Disease Patients: A Randomized, Double-Blind, Placebo-Controlled Trial

**DOI:** 10.15171/apb.2018.036

**Published:** 2018-06-19

**Authors:** Somayyeh Asghari, Maryam Rafraf, Laleh Farzin, Mohammad Asghari-Jafarabadi, Seyed-Mostafa Ghavami, Mohammad-Hossein Somi

**Affiliations:** ^1^Students’ Research Committee, Faculty of Nutrition and Food Science, Tabriz University of Medical Sciences, Tabriz, Iran.; ^2^Nutrition Research Center, Department of Community Nutrition, Faculty of Nutrition and Food Science, Tabriz University of Medical Sciences, Tabriz, Iran.; ^3^Road Traffic Injury Research Center, Tabriz University of Medical Sciences, Tabriz, Iran.; ^4^Department of Statistics and Epidemiology, Faculty of Health, Tabriz University of Medical Sciences, Tabriz, Iran.; ^5^Department of Radiology, Paramedical school, Tabriz University of Medical Sciences, Tabriz, Iran.; ^6^Liver and Gastrointestinal Diseases Research Centre, Tabriz University of Medical Sciences, Tabriz, Iran.

**Keywords:** Clinical trial, Nonalcoholic fatty liver disease, Oxidative stress, Resveratrol supplementation

## Abstract

***Purpose:*** Despite a proposed role for oxidative stress in the pathogenesis of nonalcoholic fatty liver disease (NAFLD), antioxidant approaches have not been sufficiently investigated in human NAFLD management. Resveratrol has been reported to possess a wide range of biological functions, including antioxidant activities. This study aimed to evaluate the effects of resveratrol supplementation on oxidative/anti-oxidative status in patients with NAFLD.

***Methods:*** This randomized, double-blind, placebo-controlled clinical trial was conducted on 60 patients with NAFLD (males and females) aged 20 to 60 years, and body mass index (BMI) of 25-35 kg/m2. Subjects were randomly assigned to receive a daily dose of 600 mg resveratrol (2×300 mg pure trans-resveratrol capsules; n=30) or placebo capsules (n=30) for 12 wk. Fasting blood samples, anthropometric measurements, and dietary intakes were collected for all patients at baseline and at the end of the trial. Oxidative stress was evaluated by measurement of serum malondialdehyde (MDA), oxidized low-density lipoprotein (ox-LDL), total antioxidant capacity (TAC), and erythrocyte superoxide dismutase (SOD) as well as glutathione peroxidase (GSH-Px) activities. Changes in the outcomes were analyzed using analysis of covariance (ANCOVA).

***Results:*** Resveratrol supplementation did not significantly affect neither serum MDA, ox-LDL, and TAC levels, nor erythrocyte SOD and GSH-Px activities, compared to placebo group (All P>0.05). Moreover, changes in serum levels of liver enzymes (ALT, AST, GGT, and ALP) were not significant in neither of the study groups (All P>0.05).

***Conclusion:*** Resveratrol supplementation did not modify oxidative/anti-oxidative status in patients with NAFLD.

## Introduction

Non-alcoholic fatty liver disease (NAFLD) is the most common chronic liver disease worldwide, with a prevalence rate of 6-35% in the general population, depending on the assessment methods and the study population.^1^ Higher prevalences were found among those afflicted with metabolic disorders such as obesity, type 2 diabetes, and metabolic syndrome.^1,2^ NAFLD represents a spectrum of liver diseases from simple hepatic steatosis to nonalcoholic steatohepatitis (NASH), and fibrosis, which may progress to cirrhosis and even hepatocellular carcinoma.^3^ Insulin resistance and oxidative stress are among the major important risk factors for this disease, and current therapeutic approaches to NAFLD include strategies that could modify these risk factors.^4^ Insulin resistance leads to increased lipolysis and hepatic uptake of free fatty acids (FFA) and enhances hepatic triglyceride synthesis and accumulation.^5^ The excessive offer of lipids to the mitochondria would promote production of reactive oxygen species (ROS), which contribute to lipid peroxidation and eventual oxidative stress.^6,7^ The most remarkable consequence of oxidative stress is the oxidation of lipids, proteins, DNA, and enzymes, leading to cellular damage and acceleration of cell death induced by apoptosis and necrosis.^8^ Oxidative stress is considered of primary importance in the disease progression from simple steatosis to steatohepatitis and liver damage.^9^

Lipid peroxidation and ROS can also lead to depletion of nonenzymatic antioxidants as well as antioxidant enzymes such as superoxide dismutase (SOD) and glutathione peroxidase (GSH-Px). These antioxidant defenses are particularly important because they represent the direct elimination of free radicals, providing maximum protection against oxidative damages.^10^ Multiple clinical studies have demonstrated the elevated levels of circulating products of oxidative stress and lipid peroxidation,^11^ as well as decreased antioxidant levels in patients with NAFLD.^12^

The use of compounds with antioxidant activity has been postulated to have favorable effects in prevention and treatment of complications caused by oxidative stress.^13^ Despite a proposed role of oxidative stress in the pathogenesis of NAFLD, antioxidant approaches have not been sufficiently investigated in NAFLD therapy. Resveratrol (3, 4', 5- trihydroxystilbene), a natural non-flavonoid phenolic compound found mainly in grapes, berries and red wine, has been known to have a wide array of biological functions.^14^ Most animal studies have shown that resveratrol acts as a potent antioxidant agent against oxidative stress.^15-18^ Furthermore, resveratrol has been reported to increase the activities of certain antioxidant enzymes,^15,19^ decrease lipid peroxidation products,^17,20,21^ and elevate the levels of nonenzymatic antioxidant compounds.^22^

Despite the numerous studies in rodents, to date, there have been limited clinical trials with contradictory results on the effects of resveratrol supplementation on NAFLD characteristics.^23^ Given the potential antioxidant properties of resveratrol, and the limited human research about its possible effects in NAFLD, we designed this study to investigate whether resveratrol supplementation could improve oxidative/anti-oxidative status in patients with NAFLD.

## Materials and Methods

### Study subjects

Patients aged 20 to 60 years with body mass index (BMI) ranging 25-35 kg/m^2^, were recruited from Golgasht clinic (Tabriz, Iran) between January and June 2016.

In this study, liver ultrasonography was used to diagnose NAFLD. All the trans-abdominal ultrasound measurements of liver steatosis were performed by one radiologist, at baseline and at the end of the study.

Pregnant, lactating and postmenopausal women, as well as professional athletes were excluded from the study. Other exclusion criteria also included smoking, consumption of any alcoholic beverages, suffering from inherited liver disorders, known liver, cardiovascular, kidney and gastrointestinal diseases, diabetes, thyroid dysfunction, or malignancies, using medications such as hepatotoxic drugs, corticosteroids, hormonal drugs, psychotropic and antidepressants, oral anti-diabetic and lipid-lowering drugs, taking any kinds of supplements three months prior to the study and/or during the study period.

Sample size of the study was determined based on the primary information obtained for oxidized-low density lipoprotein (ox-LDL) from Tome-Carneiro study.^24^ By considering 95% confidence, 80% power and two-tailed statistical test, the sample size was computed to be 25 per group. In order to cover possible dropouts, the sample size was increased to 30 in each group.

### Study Design and Measurements

This study was designed as a randomized, double-blind placebo-controlled clinical trial. Sixty patients (41 males and 19 females) were randomly assigned to receive a daily dose of 600 mg resveratrol (2×300 mg capsules per day of pure trans-resveratrol; n=30) or identical placebos (2×300 mg capsules per day of starch; n=30) for 12 weeks. An independent person not involved in the study process prepared both resveratrol and placebo bottles, and labeled them as A or B. The patients and researchers were all blinded to the treatment assignment until the statistical analyses were completed. Randomization was carried out using block randomization procedure of size 2 and 4, stratified by age (20-40, 40-60 years old), sex (male, female), and BMI (25-30, 30-35 kg/m^2^). Random allocation software was used for generating a random sequence, by the study statistician.

At baleline, all patients were educated about the basic principles of a healthy diet and weight control by a nutritionist, based on Food Guide Pyramid and the recommendations of the NIH Obesity Education Initiative Expert Panel. Patients’ compliance was monitored by counting the number of returned capsules every two weeks. The patients were excluded from the study if the remaining capsule counts were more than 10% of the expected ones, at the end of the study. At each visit, the patients received a supply of their medications for the next two weeks.

Anthropometric parameters were measured for all patients at baseline and at the end of the study. Height was measured without shoes with a precision of 0.1 cm. Weight was measured in light clothing by a Seca scale to the nearest 0.1 kg. BMI was calculated as weight (kg) divided by squared height (m^2^).

To assess daily dietary intakes of the patients, they were asked to complete a consecutive three-day food record questionnaire, including two weekdays and one weekend, at baseline and week 12. Average daily dietary intakes were determined by using the modified Nutritionist 4 software (First Databank, Hearst Corp, San Bruno, CA, USA). Physical activity level was also assessed by using the international physical activity questionnaire (IPAQ),^25^ at baseline and week 12.

Blood samples (10cc) were obtained after a 12-h overnight fast, at baseline and at the end of the study. The serum samples were separated from whole blood (9cc) by centrifugation at 3500 rpm for 10 min. The remaining whole blood samples (1cc) were collected and stabilized in EDTA tubes. Both serum and whole blood samples were immediately stored at -70ºC until performing the analyses. Biochemical measurements were conducted at Drug Applied Research Center (Tabriz University of Medical Sciences, Tabriz, Iran). Alanine aminotransferase (ALT), aspartate aminotransferase (AST), gamma-glutamyl transferase (GGT), and alkaline phosphatase (ALP) were measured by enzymatic colorimetric assay (Pars Azmoon Inc. kit, Iran) and autoanalyzer set (Alcyon 300, USA). Serum concentration of malondialdehyde (MDA) was determined by thiobarbituric acid reactive substances (TBARS) method described by Bilici et al.^26^ Total antioxidant capacity (TAC) was measured using spectrophotometry method, with Randox kit (Randox Laboratories, Ltd., UK). Erythrocyte SOD and GSH-Px activities were measured spectrophotometrically using a Ransod and Ransel kit, respectively (Randox Laboratories, Ltd., UK). Results were expressed as units of SOD and GSH-Px activity per milligram of hemoglobin (Hb). Hb was measured in the hemolysates by the commercial cyanmethemoglobin method (Pars Azmoon Inc. kit, Iran). Serum levels of ox-LDL were measured by enzyme-linked immunosorbent assay (ELISA; Human ox-LDL ELISA kit, Crystal Day Bio-Tec, China).

### Statistical Analysis

All statistical analyses were performed by using SPSS software version 17 (SPSS Inc., IL, Chicago, USA). All analyses were done using intention to treat (ITT) principle according to the CONSORT statement.

Kolmogorov–Smirnov test was used to determine normality of the distribution of variables. Data were presented as frequency (percent) for categorical variables, mean (standard deviation (SD)) for numeric normal variables and geometric mean (95% confidence interval) for numeric non-normal variables. Analyses of non-normal variables were performed after log transformation. Comparisons of baseline characteristics were performed using independent samples t-test and chi-squared test, where appropriate. Paired samples t-test was applied for within group comparisons. The effect of the intervention was determined by analysis of covariance (ANCOVA) adjusting for baseline values and confounders. In all analyses, P<0.05 was considered to be statistically significant.

## Results and Discussion

**Figure 1:**
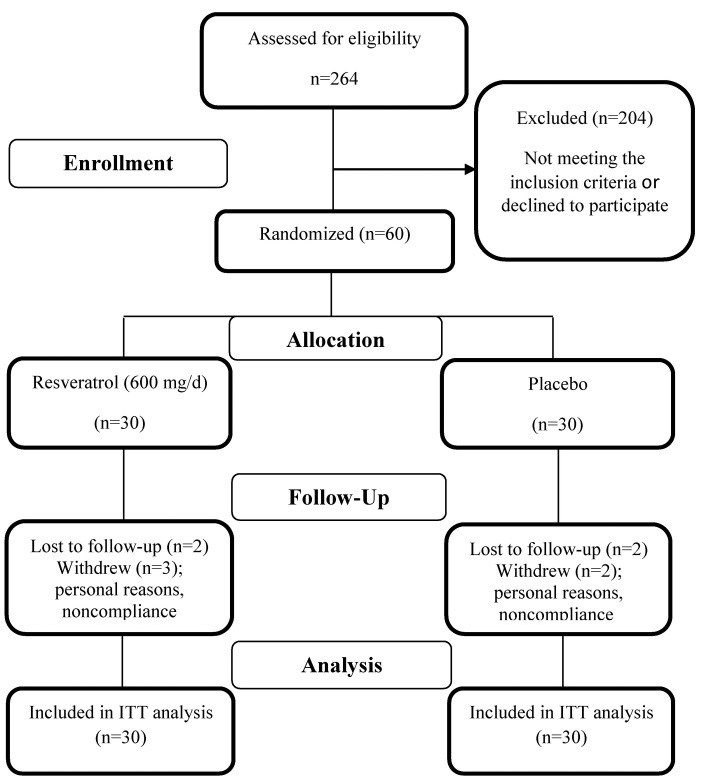
Study flow diagram

Sixty patients were enrolled in the study, and 51 (35 male and 16 female) completed the 12-week intervention: 25 in the resveratrol group and 26 in the placebo group. The reasons for the loss to follow up are described in the study flowdiagram (Figure 1). Participants' compliance was high in both study groups. Capsule counts indicated that more than 90% of the participants took more than 90% of their capsules. No adverse effects or symptoms were reported throughout the study.

Baseline characteristics of the study patients are presented in Table 1. There were no significant differences in age, sex, BMI, liver steatosis grade, or physical activity levels among the study groups at baseline. No significant changes were found in patients’ physical activity level and liver steatosis grade throughout the study neither within, nor between the two groups (P>0.05).

The total daily intakes of energy, macronutrients and known antioxidants are shown in Table 2. There were no significant differences between the two study groups in terms of mean daily intake of energy and macronutrients at baseline. Calorie and macronutrient intakes did not change significantly in neither of the groups, throughout the study (P>0.05). Changes in mean daily dietary antioxidants intakes of study subjects were not significant throughout the study neither within, nor between the two groups (P>0.05).

BMI reduced significantly in the resveratrol group compared to the placebo group (MD= -0.31, 95% CI: -0.64 to -0.02), P<0.05) (data not shown). This finding was in accord with a recent study which indicated a significant decrease in BMI of metabolic syndrome patients following administration of 500 mg resveratrol for three months.^27^ Some previous animal studies have also shown the antiobesity potential of resveratrol, which might be attributed to its effect on adipogenesis inhibition and lipolysis induction.^28,29^ Given the effect of resveratrol on improving mitochondrial biogenesis and function,^30-32^ it is probable that resveratrol increases the whole-body energy expenditure; this has been demonstrated in some rodent studies.^31,33^ However, several studies have not confirmed the beneficial effects of resveratrol on body weight^34,35^ or energy expenditure.^36,37^ Thus, the exact effects of resveratrol on anthropometric indices are still controversial.

**Table 1:** Baseline characteristics of the study patients

**Variables**	**Control** (n=30)	**Resveratrol** (n=30)	***P*** **-value**
Age (y)*	38.50 (30, 48)	40.00 (22, 58)	
**Sex, n (%)**			
Male	19 (63.3%)	21 (70.0%)	
Female	11 (36.7%)	9 (30.0%)	
BMI (kg/m^2^)**	30.41 (3.39)	30.78 (3.10)	
**PAL, n (%)**			0.43^§^
light	15 (50.0%)	11 (36.7%)	
moderate	15 (50.0%)	16 (53.3%)	
vigorous	0 (0.0%)	3 (10.0%)	
**Grade of liver steatosis**, **n (%)**			0.61^§^
grade 1	11 (42.3%)	10 (40.0 %)	
grade 2	15 (57.7%)	13 (52.0%)	
grade 3	0 (0.0%)	2 (8.0%)	

BMI: body mass index; PAL: physical activity level * Data are expressed as median (min, max) **Data are expressed as mean (standard deviation) ^§^ Fisher’s exact test

**Figure 2:**
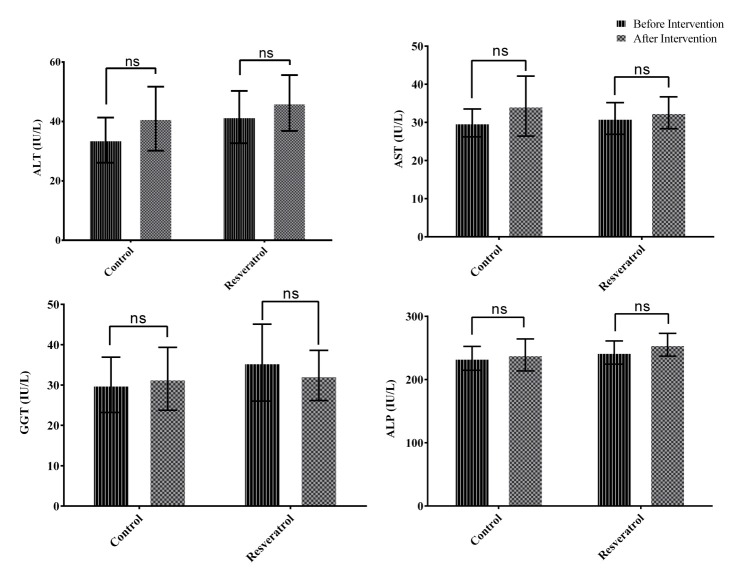
Mean (±95% Confidence interval) of liver enzymes levels in resveratrol and placebo groups after 12 weeks intervention (n=30 per group).
ALT: alanine aminotransferase, AST: aspartate aminotransferase, GGT: gamma-glutamyl transferase, ALP: alkaline phosphatase
Analysis of covariance (ANCOVA) adjusted for baseline values and mean change of BMI, daily energy intake and physical activity level showed no significant differences between resveratrol and placebo groups.

**Figure 3:**
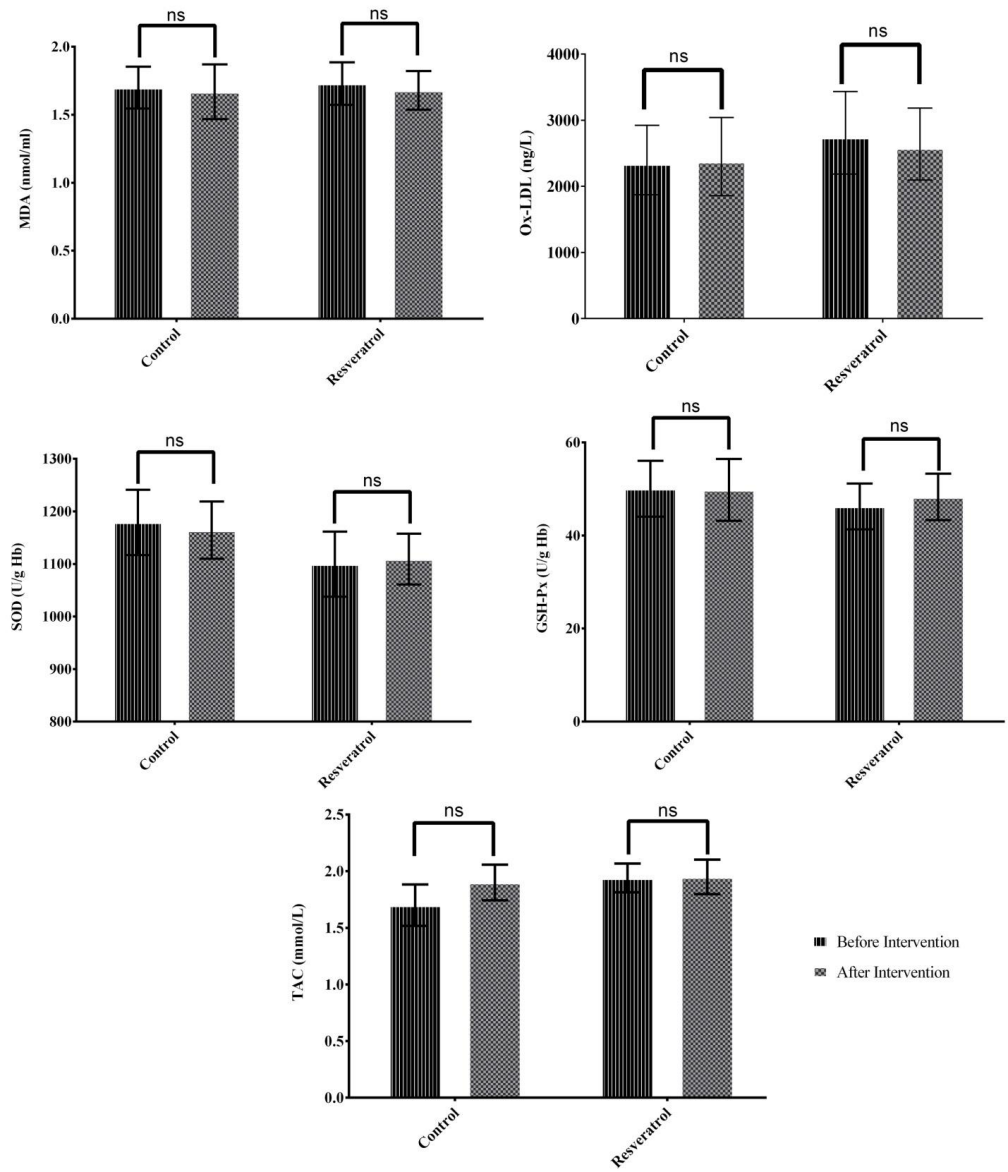
Mean (±95% Confidence interval) of oxidative stress markers in resveratrol and placebo groups after 12 weeks intervention (n=30 per group). Geometric mean (95% confidence interval) was expressed for ox-LDL.
MDA: Malondialdehyde, SOD: Superoxide Dismutase, GSH-Px: Glutathione Peroxidase, TAC: Total Antioxidant Capacity, Ox-LDL: Oxidized Low-density Lipoprotein.
Analysis of covariance (ANCOVA) adjusted for baseline values and mean change of BMI, daily energy intake and physical activity level showed no significant differences between resveratrol and placebo groups.

Biochemical characteristics of patients at baseline and after the 12 weeks intervention are presented in Figure 2 and Figure 3. There were no significant differences in biochemical values between the two study groups at baseline. Liver enzyme levels (ALT, AST, GGT and ALP) did not change significantly in resveratrol and placebo groups (All P>0.05). Results of ANCOVA also showed no significant differences in liver enzymes levels between the two groups after adjusting for the baseline values and mean change of BMI, daily energy intake and physical activity level, at the end of the study (All P>0.05; Figure 2).

Previous animal studies have shown that resveratrol protects the liver against steatosis^14,16^ and reduces intracellular lipids in the liver.^38^ However, similar to our results, Heebøll et al.^39^ reported no improvement in intrahepatic lipid content and serum levels of liver enzymes by a daily dose of 1500 mg resveratrol. Administering 3000 mg resveratrol for eight weeks, not only failed to show any significant improvements in NAFLD features, but also increased liver enzymes levels.^40^ On the other hand, Faghihzadeh et al.^41^ found significant improvements in liver steatosis and ALT levels by 500 mg resveratrol supplementation for three months, in NAFLD patients. A similar study with 600 mg of resveratrol also reported significant improvements in liver enzymes levels without any changes in liver steatosis degree.^42^ These inconsistent results may be related to the stage of the disease, method of measuring liver fat content, different dosage of resveratrol administered, or baseline metabolic characteristics of the patients. In the present study, baseline serum values of liver enzymes were in normal range in the study subjects; this might have contributed to the non-significant changes in these variables.

Following 12 weeks intervention, serum MDA, ox-LDL and TAC did not change significantly in resveratrol and placebo groups. No significant changes were observed in the activities of SOD or GSH-Px enzymes in neither of the groups (P>0.05). Results of ANCOVA adjusted for baseline values and mean changes of BMI, daily energy intake and physical activity level, showed no significant differences for serum MDA, ox-LDL and TAC as well as SOD and GSH-Px activities between resveratrol and placebo groups, at the end of the intervention (All P>0.05; Figure 3).

Extensive evidence has shown that oxidative stress may be one of the key mediators of hepatic injury in NAFLD and play a fundamental role in the progression of simple steatosis towards NASH.^9^

Elevated systemic levels of oxidative stress biomarkers and lipid peroxidation, as well as decreased antioxidant levels, have been found in patients with NAFLD.^11,12^

MDA, a common marker of lipid peroxidation, is generally produced by ROS interaction with polyunsaturated fatty acids, and used to evaluate the oxidative stress status.^8^ Ox-LDL is another common serum biomarker of oxidative stress and lipid peroxidation, which we measured. High ox-LDL level may play a major role in atherosclerosis and cardiovascular disease.^43^

In the present study, supplementation with 600 mg of resveratrol for 12 weeks, had no beneficial effects on serum MDA levels. Serum ox-LDL levels did not change significantly, either. To our knowledge, no intervention study has been carried out previously among NAFLD patients, to investigate the levels of these oxidative markers after resveratrol supplementation. In a study on normal-weight healthy subjects performed by Ghanim et al.,^44^ a Polygonum cuspidatum extract containing 40 mg resveratrol, induced a significant reduction in ROS generation in mononuclear cells. Schmatz et al.,^8^ demonstrated that resveratrol treatment prevented the elevation in TBARS levels in kidney and liver of diabetic rats. In another study on fructose-fed rats, resveratrol induced a significant reduction in TBARS levels, indicating a possible role of resveratrol in free radical inactivation.^45^

**Table 2:** Daily dietary intakes of energy, macronutrients and known antioxidants of the study patients at baseline and after the 12-wk intervention

**Variables**		**Control** (n=30)	**Resveratrol** (n=30)	**P-value**
**Energy (Kcal/d)**	Before	2082.6 (246.2)	2154.4 (307.8)	0.36^†^
	After	2068.6 (218.8)	2143.7 (355.1)	0.76^‡^
	P-value^§^	0.17	0.82	
**Carbohydrate (g/d)**	Before	321.4 (47.8)	336.1 (58.2)	0.33^†^
	After	318.0 (34.7)	328.0 (55.4)	0.87^‡^
	P-value^§^	0.32	0.28	
**Protein (g/d)**	Before	83.2 (5.7)	84.8 (12.0)	0.52^†^
	After	85.3 (9.8)	87.7 (16.7)	0.48^‡^
	P-value^§^	0.11	0.12	
**Total fat (g/d)**	Before	52.8 (3.9)	53.6 (6.3)	0.59^†^
	After	53.4 (4.9)	54.6 (10.9)	0.85^‡^
	P-value^§^	0.10	0.62	
**Vitamin E (mg/day)**	Before	10.53 (4.32)	11.11 (5.68)	0.66^†^
	After	8.96 (6.56)	10.05 (5.95)	0.50^‡^
	P-value^§^	0.28	0.48	
**Vitamin C (mg/day)**	Before	86.42 (38.69)	85.53 (38.55)	0.93^†^
	After	90.33 (42.76)	90.87 (40.90)	0.96^‡^
	P-value^§^	0.71	0.61	
**β-Carotene (µg/day)**	Before	566.17 (231.45)	582.16 (264.13)	0.80^†^
	After	623.14 (244.20)	630.27 (264.33)	0.91^‡^
	P-value^§^	0.36	0.48	
**Zinc (mg/day)**	Before	8.09 (3.03)	7.68 (3.31)	0.61^†^
	After	8.28 (3.13)	7.92 (3.09)	0.66^‡^
	P-value^§^	0.81	0.77	
**Selenium (µg/day)**	Before	67.33 (28.50)	65.42 (31.09)	0.81^†^
	After	67.87 (27.07)	65.24 (28.56)	0.72^‡^
	P-value^§^	0.94	0.98	

Data are expressed as mean (standard deviation) ^†^ Independent t-test ^‡^ Analysis of covariance (ANCOVA) adjusted for baseline values ^§^ Paired t-test

There is increasing evidence that antioxidant-rich dietary patterns, induce significant reductions in LDL oxidation.^46^ A recent trial performed in hypercholesterolemic patients demonstrated that resveratrol at a dose of 8 mg/d significantly reduced serum ox-LDL during 6 months.^24^ The evidence suggests that resveratrol exerts its antioxidant activities by scavenging ROS and reactive nitrogen species (RNS) and secondary organic radicals formed as a result of the reaction of biomolecules with ROS and RNS.^47^

The increased lipid peroxidation might be explained by increased oxidative stress as a result of the antioxidant depletion.^48^ LDL and other biomolecules are protected from oxidative damage by the action of blood antioxidant capacity.^49^ TAC is an indicator of the overall protection of antioxidants against oxidative stress in body fluids and cell components.^50^ Plasma antioxidant status includes enzymatic and nonenzymatic antioxidant activities.^49^ Enzymatic antioxidants like SOD and GSH-Px are the first line of defense against ROS and play a vital role in scavenging free radicals. SOD catalyzes the conversion of superoxide anion radical to hydrogen peroxide (H_2_O_2_). H_2_O_2_ is then degraded to H_2_O by the action of GSH-Px, which is one of the body’s most potent defenses; it protects cells from damage caused by lipid peroxide radicals and H_2_O_2_.^48^ A decrease in the action of these antioxidant enzymes may lead to an excessive levels of free radicals and H_2_O_2_ and consequently increased hydroxyl radicals generation, which results in further initiation and promotion of lipid peroxidation.^8^

Resveratrol supplementation in our study did not affect erythrocyte SOD and GSH-Px activities. TAC did not change significantly, either. Similar to our findings, Chachay et al.,^40^ found no changes in SOD and GSH-Px activities and TAC serum levels after eight-week administration of 3000 mg/d resveratrol in patients with NAFLD. However, in a randomized, double-blind, cross-over trial on healthy adult smokers, 500 mg resveratrol/d for 30 days increased TAC values.^51^

Some rodent studies have demonstrated the effects of resveratrol on regulation of oxidative stress. Zhu et al.,^20^ showed decreased ROS levels and increased glutathione (GSH), GSH-Px and SOD levels after resveratrol treatment in KKAy mice, suggesting that resveratrol has a favorable anti-oxidative effect. Schmatz et al.,^8^ also demonstrated that resveratrol treatment (10 and 20 mg/kg) prevented the reduction in SOD activity in liver and kidney of streptozotocin-induced diabetic rats. In addition, it has been reported that resveratrol could upregulate mRNA expression of antioxidant enzymes.^52,53^

The main finding of our study was that resveratrol supplementation is not associated with detectable physiological anti-oxidative effects in subjects with NAFLD. In this study, there were no significant differences in daily dietary intakes of macronutrients and known antioxidants between the two groups, by the end of the study. Therefore, dietary factors are not likely to have confounded our results. It is probable that non-significant changes in oxidative stress markers are partly attributed to the clinical and metabolic characteristics of our studied subjects. The severity of existing metabolic abnormalities before treatment seem to be of importance for measurable metabolic benefits of resveratrol supplementation.^54,55^ It is possible that the subjects in the present study were relatively ‘metabolically healthy’ in terms of oxidative/antioxidative status, and were therefore unlikely to experience any improvements after resveratrol supplementation.

Another important aspect to be discussed is the dose of resveratrol administered, which may contribute to its antioxidant properties. In the study by Brasnyó et al.,^56^ daily dose of 10 mg of resveratrol for four weeks, significantly decreased urinary ortho-tyrosine excretion as a measure of oxidative stress in type 2 diabetic patients. Hassan-Khabbar et al.^57^ reported that resveratrol with a dose of 0.2 mg/kg in rats, increased glutathione reductase, SOD, and catalase activities; however, at a high dose (20 mg/kg), became pro-oxidant with a reduced GSH levels and antioxidant enzyme activities. Such pro-oxidant action of resveratrol has been proposed to be involved in anticancer mechanisms of polyphenols.^19^ It has been also proposed that resveratrol may act as a pro-oxidant during low oxidative conditions; whereas, it could work as an antioxidant under strong oxidative status.^58^ Therefore, its function seems to depend on the dose, different pathological conditions, environmental factors, nutritional status as well as possible interactions with other compounds.^58-60^ The differences in bioavailability of various formulations, together with the duration of treatment periods, and metabolic characteristics of the patients included in the studies might also influence the study results. However, the cellular mechanisms linking resveratrol and oxidative stress are not fully understood and remain to be determined.

The current study had several strengths including a high compliance rate and the randomized double-blind, placebo-controlled design which minimized the potential bias and confounding effects of factors other than the study supplement. Use of the liver ultrasound technique as the diagnostic criteria for NAFLD was one of the limitations of our study. However, ultrasonography is currently the preferred non-invasive, inexpensive and widely available method for screening fatty liver in clinical and population-based studies, and is regarded as the first-line diagnostic tool. It should also be noted that the findings of this study are not generalizable to other studies concerning different doses and durations of resveratrol supplementation.

## Conclusion

This trial showed that supplementation with 600 mg of resveratrol could not modify oxidative/anti-oxidative status in patients with NAFLD. Further human research is needed to explore potential antioxidant properties of resveratrol in NAFLD, and to elucidate the optimum therapeutic dose.

## Acknowledgments

We thank the Research Vice-Chancellor and Nutrition Research Center of Tabriz University of Medical Sciences, Tabriz, Iran, for the financial support. We also appreciate the patients who kindly participated in this study. This article was written based on data set of PhD thesis registered in Tabriz University of Medical Sciences, Iran.

## Ethical Issues

The study protocol was approved by the Ethics committee of Tabriz University of Medical Sciences (TBZMED.REC.1394.823) and conducted according to the principles of the Declaration of Helsinki. Informed consent was obtained from participants prior to the study.

## Conflict of Interest

The authors report no declarations of interest.
